# Hepatitis C virus core protein potentiates proangiogenic activity of hepatocellular carcinoma cells

**DOI:** 10.18632/oncotarget.21407

**Published:** 2017-09-30

**Authors:** Yu-Yun Shao, Min-Shu Hsieh, Han-Yu Wang, Yong-Shi Li, Hang Lin, Hung-Wei Hsu, Chung-Yi Huang, Chih-Hung Hsu, Ann-Lii Cheng

**Affiliations:** ^1^ Graduate Institute of Oncology, College of Medicine, National Taiwan University, Taipei City, Taiwan; ^2^ Department of Pathology, Graduate Institute of Pathology, College of Medicine, National Taiwan University, Taipei City, Taiwan; ^3^ Department of Oncology, National Taiwan University Hospital, Taipei City, Taiwan; ^4^ Department of Pathology, National Taiwan University Hospital, Taipei City, Taiwan; ^5^ Department of Internal Medicine, National Taiwan University Hospital, Taipei City, Taiwan; ^6^ National Taiwan University Cancer Center, Taipei City, Taiwan

**Keywords:** angiogenesis, core protein, hepatitis C virus, hepatocellular carcinoma

## Abstract

Increased angiogenic activity has been demonstrated in hepatitis C virus (HCV)-related hepatocellular carcinoma (HCC), but the mechanism was unclear. To study the role of HCV core protein, we used tube formation and Matrigel plug assays to assess the proangiogenic activity of an HCC cell line, HuH7, and 2 of its stable clones—HuH7-core-high and HuH7-core-low, with high and low HCV core protein expression, respectively. In both assays, HuH7-core-high and HuH7-core-low cells dose-dependently induced stronger angiogenesis than control cells. HuH7 cells with HCV core protein expression showed increased mRNA and protein expression of vascular endothelial growth factor (VEGF). VEGF inhibition by bevacizumab reduced the proangiogenic activity of HuH7-core-high cells. The promotor region of *VEGF* contains the binding site of activator protein-1 (AP-1). Compared with controls, HuH7-core-high cells had an increased AP-1 activity and nuclear localization of phospho-c-jun. AP-1 inhibition using either RNA knockdown or AP-1 inhibitors reduced the VEGF mRNA expression and the proangiogenic activity of HuH7-core-high cells. Among 131 tissue samples from HCC patients, HCV-related HCC revealed stronger VEGF expression than did hepatitis B virus-related HCC. In conclusion, increased VEGF expression through AP-1 activation is a crucial mechanism underlying the proangiogenic activity of the HCV core protein in HCC cells.

## INTRODUCTION

Most patients with hepatocellular carcinoma (HCC) have known etiological factors, such as hepatitis B virus (HBV), hepatitis C virus (HCV), and alcoholic liver disease [1-3]. Different etiological factors may cause diverse carcinogenic processes and result in various biological behaviors. One of the unique biological behaviors of HCV-related HCC is the increased angiogenesis. By evaluation of microvessel density in HCC tumor samples from patients with HBV or HCV infection, HCV-related HCC has been demonstrated to exhibit higher angiogenic activity [4]. In an *in vitro* study, the conditioned medium collected from HCV-infected HCC cells induced more angiogenesis in the chick chorioallantoic membrane assay than that from uninfected HCC cells did [5].

The actual molecular mechanism underlying the higher angiogenic activity of HCV-related HCC remains unclear. Previous studies revealed different mechanisms in various experimental models, such as stabilization of hypoxia-inducible factor (HIF)-1α by the subgenomic replicon of HCV [5, 6], increased activity of Jun amino-terminal kinases (JNK), mitogen-activated protein kinase (MAPK), or androgen receptor pathways by the HCV core protein [7, 8], and increased angiopoietin (ANG)-2 expression by the HCV infection [9]. No single study has thus far demonstrated that HCV can induce angiogenic activity of HCV-related HCC cells through regulating angiogenic pathways in one experimental setting.

Several components of HCV were reported to have impact on HCC cells in hepatocarcinogenesis, including the envelope protein E2, the nonstructural protein NS5A, and the core protein [9-13]. Among them, the HCV core protein has the strongest potential associations with angiogenesis because it has been reported to increase the expression of angiogenic factors, including vascular endothelial growth factor (VEGF) and ANG-2 [9, 11-13]. In addition, HCV core protein has also been reported to enhance metastasis and epithelial–mesenchymal transition of HCC cells [14, 15]. Thus, this study aimed to examine the mechanisms how the HCV core protein induces the proangiogenic activity of HCC cells.

## RESULTS

### HCV core protein increases the proangiogenic activity of HCC cells

**Figure 1:**
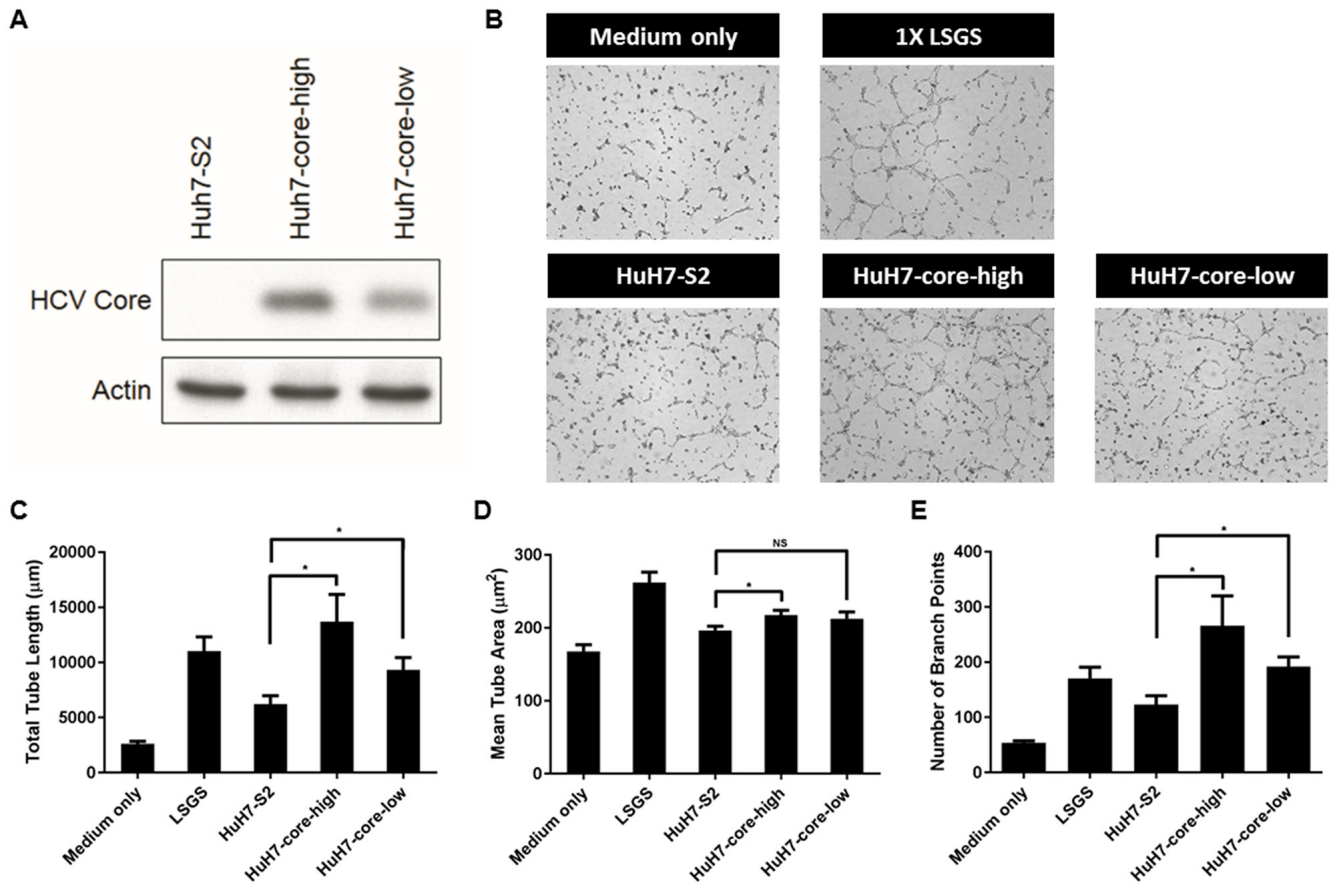
**(A)** Western blot results showing the HCV core protein expression level in HuH7-S2, HuH7-core-high, and HuH7-core low cells. **(B-E)** Tube formation assay. (B) Images (50×) displaying the effect of controlled media of 3 HCC cell lines or control media on the tube formation of HUVECs. Quantitative results of the tube formation assay showed the total tube length (C), mean tube area (D), and numbers of branch points (E). Data are presented as mean ± SEM. Low serum growth supplement (LSGS) was the positive control. *: *p* < 0.05; NS = not significant.

We used a lentivirus-based vector, S2, to overexpress the HCV core protein (genotype Ib) in HuH7 cells [16]. Two stable clones with HCV core protein expression, HuH7-core-high and HuH7-core-low, were established. HuH7-core-high and HuH7-core-low cells expressed high and low levels of the HCV core protein, respectively (Figure 1A). Another stable clone with the empty vector, HuH7-S2 cells, served as the control and showed no expression of the HCV core protein (Figure 1A). The tube formation assay showed that both HuH7-core-high and HuH7-core-low cells induced more potent angiogenesis than HuH7-S2 cells did (Figure 1B): HuH7-core-high cells induced angiogenesis with greater total tube lengths (*p* = 0.02), greater mean tube areas (*p* = 0.04), and more branch points (*p* = 0.03) (Figure 1C-1E), and HuH7-core-low cells induced angiogenesis with greater total tube lengths (*p* = 0.05) and more branch points (*p* = 0.01) (Figure 1C-1E). The potentiation of the proangiogenic activity of HuH7 cells induced by the HCV core protein was dose dependent, as demonstrated through the more potent angiogenesis induced by HuH7-core-high cells than by HuH7-core-low cells (Figure 1C-1E).

**Figure 2:**
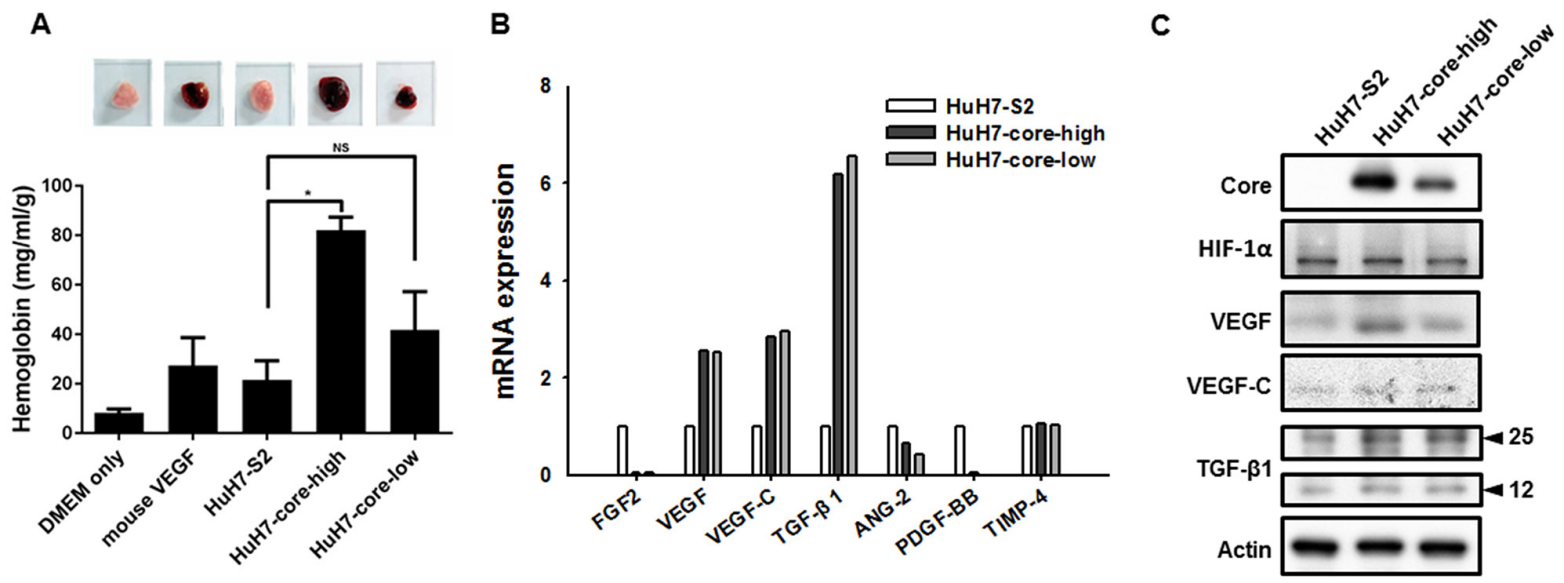
**(A)** Matrigel plug assay. Matrigel mixed with DMEM only, DMEM + mouse VEGF, or culture medium of HuH7-S2, HuH7-core-high, or HuH7-core-low cells was subcutaneously injected into 5-week-old mice. The Matrigel plugs were harvested 7 days later, and their hemoglobin content was evaluated. Data are presented as mean ± SEM. *: *p* < 0.05; NS = not significant. **(B)** mRNA expression levels of genes associated with angiogenesis in HuH7-S2, HuH7-core-high, and HuH7-core-low cells were examined through quantitative reverse transcription PCR analysis with GAPDH as the internal control. The value of the HuH7-S2 cells was used as a reference to show the expression level of HuH7-core-high and HuH7-core-low cells. *: *p* < 0.05 compared to HuH7-S2 cells. **(C)** Protein expression levels of angiogenic factors in HuH7-S2, HuH7-core-high, and HuH7-core-low cells, examined through Western blot analysis.

Through the Matrigel plug assay, we confirmed that HCV core protein expression potentiated the *in vivo* angiogenesis activity of HCC cells. Seven days after the Matrigel plugs containing the culture media of HCC cells were implanted in mice, the plugs containing the culture media of HuH7-core-high and HuH7-core-low cells exhibited higher blood content than the plugs containing HuH7-S2 cell culture media did (Figure 2A). A quantitative analysis confirmed this finding and revealed a similar dose-dependent effect of the HCV core protein on the hemoglobin content, a surrogate indicator of angiogenesis.

### HCV core protein is associated with increased VEGF expression

We examined the mRNA and protein expression of several angiogenic factors to determine the factors that contributed to the proangiogenic activity of the HCV core protein. Although the mRNA expression of VEGF, VEGF-C, and transforming growth factor-β1 was increased in HuH7-core-high and HuH7-core-low cells (Figure 2B), only the protein expression of VEGF was increased in HuH7-core-high and HuH7-core-low cells (Figure 2C). Expression of HIF-1α was also similar among the 3 cell lines.

### HCV core protein-induced proangiogenic activity is reduced through VEGF inhibition

**Figure 3:**
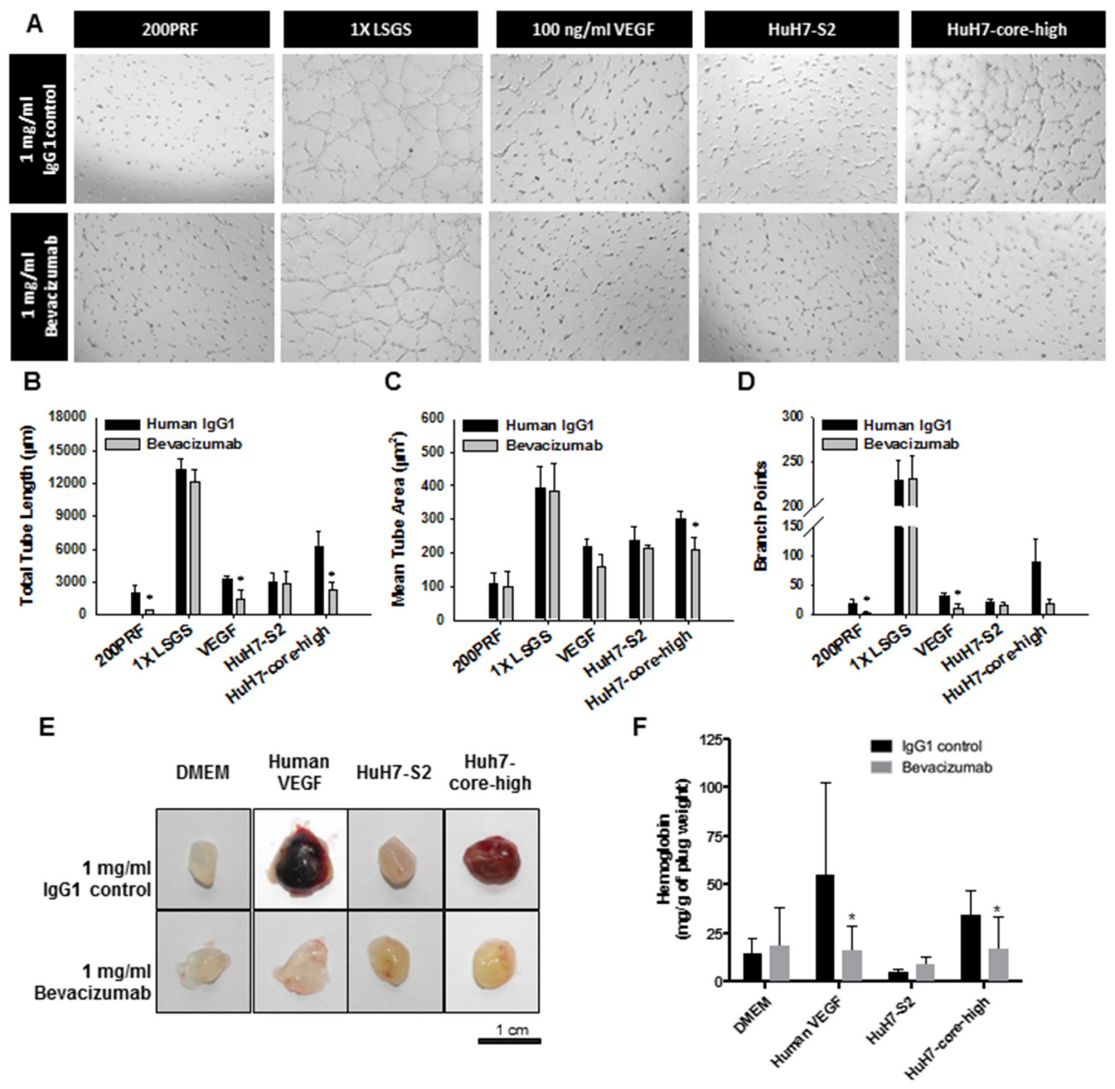
Validation of the role of VEGF in the proangiogenic activity of HuH7 cells expressing the HCV core protein through **(A-D)** the tube formation assay and **(E-F)** the Matrigel plug assay. (A) Images (50×) displaying the influence on tube formation of HUVECs by various HCC cells or control media, with control IgG1 or bevacizumab, an anti-VEGF antibody. (B-D) Quantitative results of the tube formation assay showing the total tube length (B), mean tube area (C), and numbers of branch points (D). Data are presented as mean ± SEM. Low serum growth supplement (LSGS) was the positive control. *: *p* < 0.05 compared to IgG control. (E) Images displaying the Matrigel plugs harvested 7 days after transplantation. (F) Quantitative analysis of the Matrigel plug assay results through hemoglobin quantification. Matrigel was mixed with the media as noted in the figure with control IgG1 or bevacizumab. Data are presented as mean ± SEM. Human VEGF was the positive control. *: *p* < 0.05 compared to IgG control.

To prove the increased expression of VEGF mediated the proangiogenic activity of the HCV core protein, we evaluated the effect of bevacizumab, a clinically useful monoclonal antibody blocking human VEGF, in HuH7 cells. In both the tube formation (Figure 3A-3D) and Matrigel plug (Figure 3E and 3F) assays, inhibition of VEGF by bevacizumab significantly reduced the *in vitro* and *in vivo* proangiogenic activity of HuH7-core-high cells. By contrast, the effect of VEGF inhibition on the proangiogenic activity of HuH7-S2 cells was minimal.

### High activator protein-1 activity is associated with increased VEGF expression in HCV core protein- expressing HCC cells

**Figure 4:**
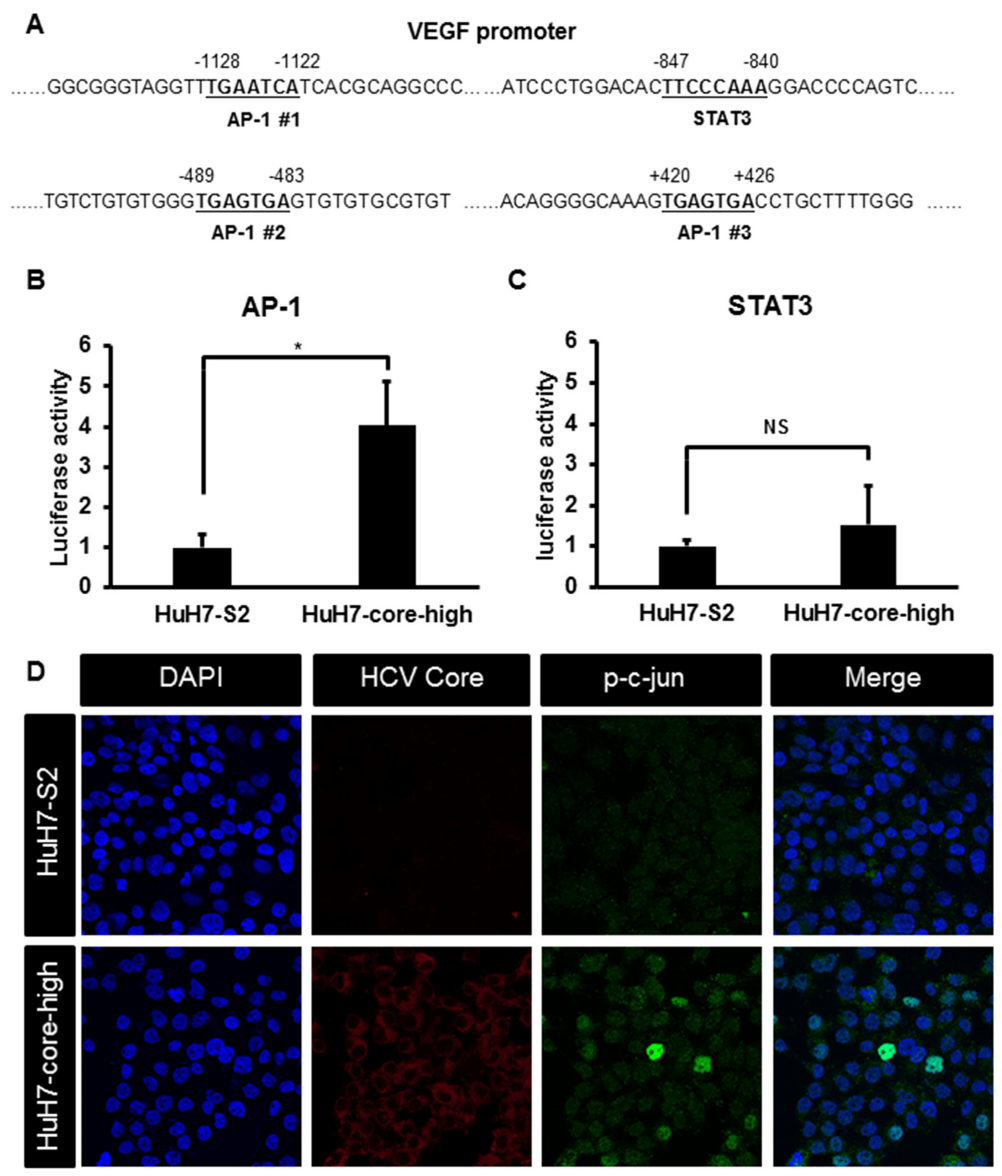
**(A)** Sequence of the VEGF promoter region showing the binding sites for AP-1 and STAT3. **(B-C)** Reporter assays showing the activity of transcription factors AP-1 (B) and STAT3 (C) in HCC cells. Data are presented as the relative luciferase activity (mean ± SEM) compared with that of HuH7-S2 cells. *: *p* < 0.05; NS = not significant. **(D)** Images (200×) of immunofluorescence staining results of HuH7-S2 and HuH7-core-high cells with DAPI for nuclear staining (blue) and antibodies against HCV core protein (red) and p-c-jun (green).

**Figure 5:**
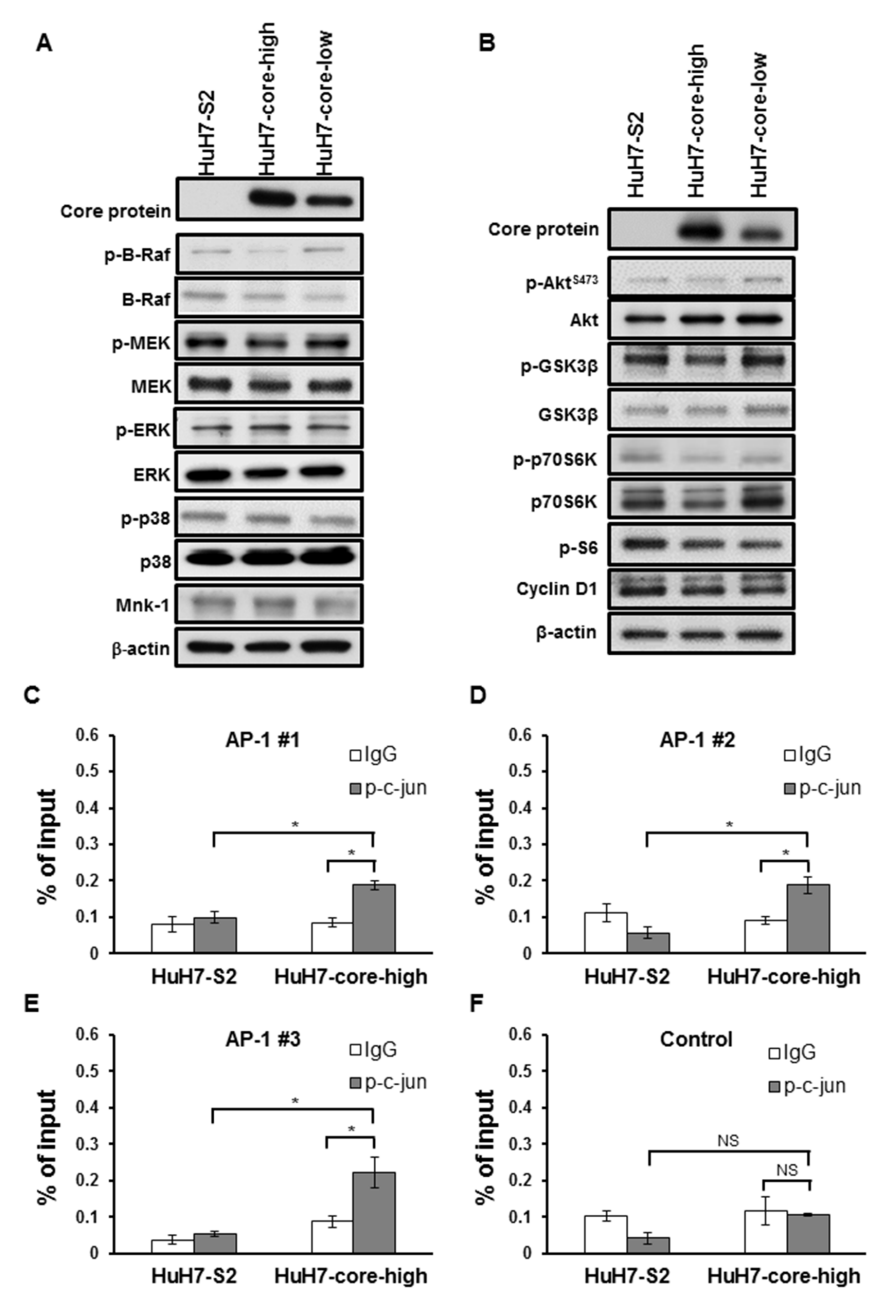
**(A-B)** Western blots showing the expression of proteins associated with the MAPK (E) and the PI3K (F) pathways in HCC cells. **(C-F)** Chromatin immunoprecipitation assay using control IgG or the anti-p-c-jun antibody. We compared the DNA fragments harboring AP-1 binding sites (C-E) and those without (F) after enrichment with control IgG or anti-p-c-jun antibody. The total DNA input amount was used as the reference. All data are presented as mean ± SEM. *: *p* < 0.05; NS = not significant.

To identify the potential mechanisms underlying increased VEGF mRNA and protein expression in HuH7 cells expressing the HCV core protein, we examined the promoter region of VEGF and observed that this promoter region contains binding sites for several transcription factors, including activator protein-1 (AP-1) and signal transducer and activator of transcription 3 (STAT3) (Figure 4A). AP-1 activity was significantly increased in HuH7-core-high cells compared with HuH7-S2 cells (Figure 4B), but STAT3 activity was similar between HuH7-S2 and HuH7-core-high cells (Figure 4C). Immunofluorescence staining showed that HuH7-core-high cells had higher expression and more nuclear localization of phospho-c-jun (p-c-jun) (Figure 4D) and phospho-c-fos (p-c-fos) (Supplementary Figure 1), which constitute the AP-1 heterodimer, than HuH7-S2 cells had. By contrast, we found no significant variations in the MAPK and PI3K/Akt pathways among the HuH7-S2, HuH7-core-high, and HuH7-core-low cell lines (Figure 5A and 5B).

The chromatin immunoprecipitation assay confirmed the binding of AP-1 to the promoter region of VEGF. In HuH7 cells expressing HCV core protein, immunoprecipitation with anti-p-c-jun antibody enriched the DNA fragments of the VEGF promoter harboring the AP-1 binding sites (Figure 5C-5E), but not the fragments without the AP-1 binding site (Figure 5F). Such enrichment was not found in control HuH7 cells (Figure 5C-5E).

### Inhibition of AP-1 activity downregulates VEGF expression and inhibits the proangiogenic activity of HCV core protein in HCC cells

**Figure 6:**
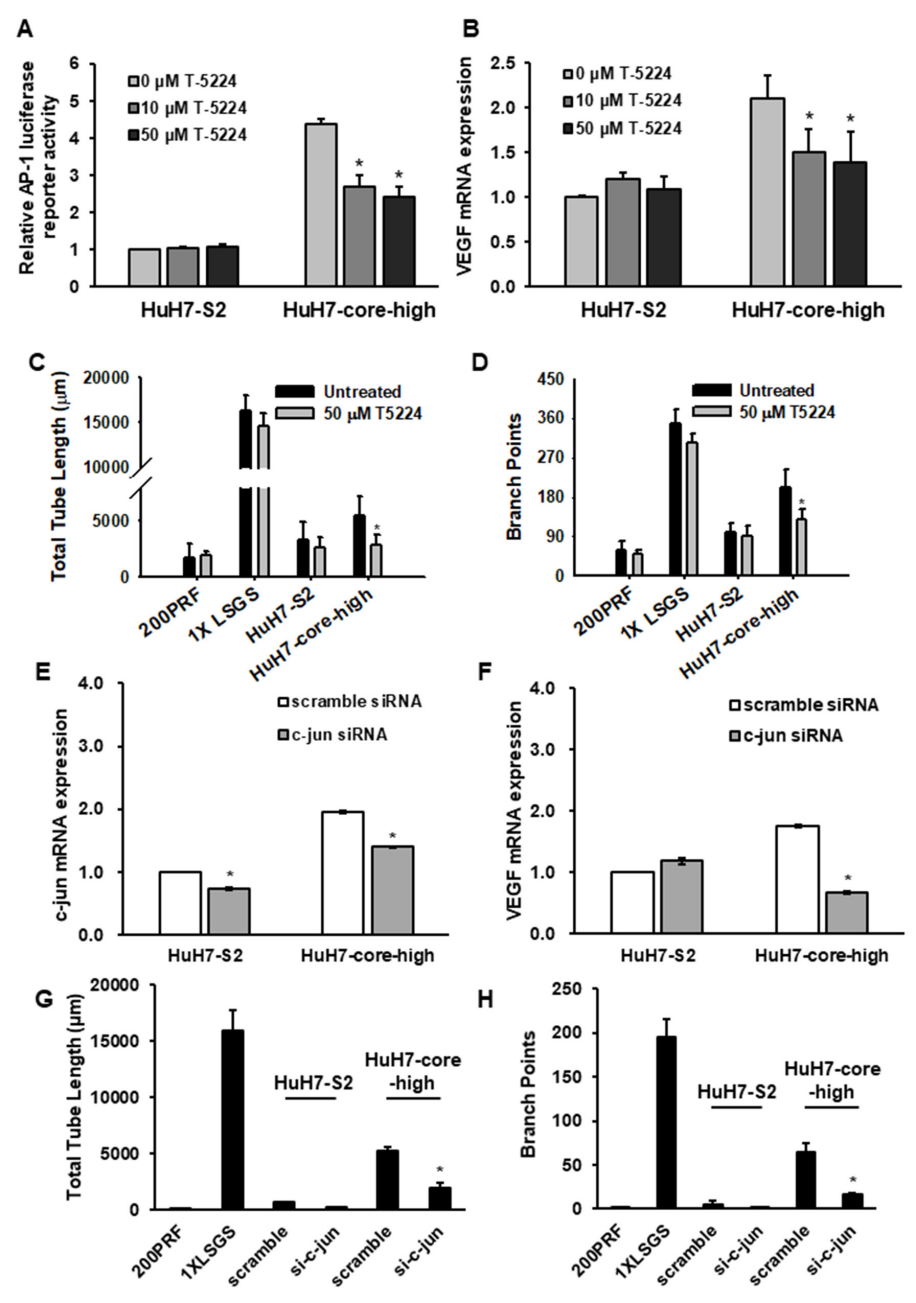
**(A)** Reporter assays displaying AP-1 activity in HuH7-S2 and HuH7-core-high cells after treatment with T-5224, an AP-1 inhibitor. The luciferase activity of HuH7-S2 cells treated with 0 μM T-5224 was the reference. **(B)** mRNA expression levels of VEGF in HuH7-S2 and HuH7-core-high cells after treatment with various concentrations of T-5224 with GAPDH as the internal control. The level in HuH7-S2 cells treated with 0 μM T-5224 was the reference. **(C-D)** Tube formation assays showing the influence of T-5224 on HCC cell lines measured by the total tube length (C) and numbers of branch points (D). **(E-F)** mRNA expression levels of c-jun (E) and VEGF (F) in HuH7-S2 and HuH7-core-high cells after knockdown of c-jun expression with siRNA. **(G-H)** Tube formation assays showing the influence of siRNA-mediated c-jun downregulation on HCC cells measured by the total tube length (G) and numbers of branch points (H). All data are presented as mean ± SEM. Low serum growth supplement (LSGS) was the positive control in the tube formation assay. *: *p* < 0.05 compared to no T-5224 treatment or scrambled siRNA.

To further verify the role of AP-1 in HCV core protein-mediated VEGF upregulation and proangiogenesis, we used the AP-1 inhibitor T-5224 to reduce AP-1 activity in the HuH7-core-high cells (Figure 6A). The increased mRNA expression of VEGF in HuH7-core-high cells was reduced by AP-1 inhibition (Figure 6B) in a concentration of T-5224 with limited effect on cell viability (Supplementary Figure 2). In tube formation assay, the proangiogenic activity of HuH7-core-high cells was reduced by T-5224 treatment, whereas the proangiogenic activity of HuH7-S2 cells was not influenced by T5224 treatment (Figure 6C and 6D). Using RNA interference knocking down c-jun expression, we also observed that the VEGF mRNA expression (Figure 6E and 6F) and the proangiogenic activity, assessed by the tube formation assay, of HuH7-core-high cells were significantly reduced (Figure 6G and 6H).

### HCV-related HCC tissues exhibited high expression of VEGF

We analyzed HCC tumor samples from 131 patients (94 with HBV-related HCC and 37 with HCV-related HCC) with matching tumor stages, macrovascular invasion, extrahepatic metastasis, and α-fetoprotein levels. HCV-related HCC was more likely to show strong VEGF staining than HBV-related HCC (95% vs 72%, *p* = 0.005). VEGF staining analysis using H scores also revealed that HCV-related HCC has stronger VEGF expression than HBV-related HCC (mean H score, 289.5 vs 248.8, *p* < 0.001; Supplementary Table 1). High p-c-jun nuclear localization was associated with high VEGF expression (*p* = 0.038; Supplementary Table 1). Representative staining results are demonstrated in Supplementary Figure 3.

## DISCUSSION

This study revealed that the HCV core protein significantly potentiates the proangiogenic activity of HCC cells both *in vitro* and *in vivo*. Our *in vitro* data suggested that the proangiogenic activity of the HCV core protein is dose-dependent and the mechanism involves increased expression of VEGF through AP-1 activation. In tumor samples, the expression of VEGF was higher in HCV-related HCC than in HBV-related HCC. To the best of our knowledge, this is the first single study to simultaneously demonstrate the increased expression of angiogenic factors and the proangiogenic activity of HCC cells by overexpressing the HCV core protein.

We used 2 assays to evaluate the angiogenic activity: the Matrigel plug assay is an *in vivo* assay that uses the hemoglobin concentration as a surrogate marker of vascular density, whereas the tube formation assay offers a direct observation of microvessels but neglects the effect of tissues and cells other than cancer cells. Although both assays have limitations, they displayed similar results showing that HCV core protein dose-dependently increases the proangiogenic activity of HCC cells. These data collectively suggest that the HCV core protein potentiates the proangiogenic activity of HCC cells by inducing secretory proangiogenic factors in HCC cells. We demonstrated that VEGF expression was increased in HCV core protein-expressing HCC cells. However, additional studies are warranted to determine whether other angiogenic factors, such as VEGF-C and ANGs, are involved.

Previously, several growth factor signaling pathways, such as the MAPK, PI3K, and JNK pathways, have been reported to involve in HCV core protein-induced VEGF expression [7, 17-20]. However, the results on the effect of the HCV core protein on the activity of the MAPK pathway have been conflicting [21-23]. In the present study, we could not identify significant changes in the MAPK or PI3K pathway on the basis of the HCV core protein expression in HCC cells. Nevertheless, we observed increased nuclear localization of p-c-jun and increased activity of AP-1, which locates downstream of the JNK pathway in HCV core protein-expressing HCC cells. In addition, we also demonstrated the binding of AP-1 to the VEGF promoter and that AP-1 inhibition reduced the high expression levels of VEGF and the proangiogenic activity of HCV core protein-expressing HCC cells. Our observation is consistent with previous reports of the AP-1 activity involved in VEGF expression [24, 25] and those of the AP-1 activity induced by HCV core protein [23, 26, 27].

Kanda et al. reported that HCV core protein activated STAT3 and then enhanced androgen receptor-mediated transcription [8]. HCV-infected HuH7 cells exhibited higher VEGF expression in the presence of androgen receptor activation, and the culture medium of these cells increased tube formation of human coronary microvascular endothelial cells. In our study, we did not find increased STAT3 activity in HCC cells expressing HCV core protein. The HCV core protein expressed in our study was geneotype Ib, but the one in the aforementioned study was genotype Ia. We used lentivirus for stable HCV core protein expression, which also differs from the previous study. These variations may result in the different findings about STAT3 activity. Further studies are warranted to elucidate the interactions among HCV core protein, androgen receptor, STAT3, and AP-1 in regulating VEGF expression and proangiogenesis in HCC cells.

Although we demonstrated that the HCV core protein was associated with the proangiogenic activity of HCC cells, other components of HCV may also be involved. For example, the envelope protein E2 has been reported to upregulated matrix metalloproteinase-2 [10], and the nonstructural protein NS5A has been reported to increase the expression of cyclooxygenase-2 [11]. These proteins may also contribute to the increased angiogenic activity in HCV-related HCC.

This study has other limitations. We did not transfect the entire virus because HCC cells allowing HCV replication might be substantially different from other HCC cell lines. For example, HuH7.5 cells were reported to be very resistant to sorafenib [28]. As a result, we could not elucidate the potential interactions between HCV core protein and other HCV components. We only used HCV core protein of HCV genotype Ib in this study because genotype Ib is the most common HCV genotype in Taiwan, which could be identified in up to 77% of HCV infected patients [29, 30]. Other genotypes of HCV may exhibit activity in regulating VEGF expression in HCC cells [8]. In addition, only HuH7 cell lines were examined in this study; whether the finding of the current study could be extended to other HCC cells remains to be proven. However, we had two independent stable clones confirming the findings that HCV core protein increased the proangiogenic activity of HCC cells.

Sorafenib, the current standard first-line therapeutic agent for advanced HCC, exhibits antiangiogenic activity. A subgroup analysis of a phase III trial revealed that sorafenib has higher efficacy in patients with HCV infection than in those with other HCC etiologies [31]. A recent meta-analysis based on 4 phase III clinical trials using sorafenib as the first-line therapy for advanced HCC revealed that HCV-positive patients gained more survival benefits than HCV-negative patients did [32]. The finding of the current study (i.e., the HCV core protein induces the expression of VEGF and promotes the proangiogenic activity of HCC cells) cannot completely clarify why sorafenib, but not other inhibitors of VEGF receptors such as sunitinib and brivanib, confers more therapeutic efficacy in HCV-positive HCC patients than in HCV-negative HCC patients [33, 34]. The reason that HCV affects the efficacy of sorafenib more than that of other agents warrants further investigation.

In conclusion, our data demonstrated that increased VEGF expression is a crucial mechanism underlying the proangiogenic activity of the HCV core protein in HCC cells, and AP-1 activation contributes to the increased VEGF expression.

## MATERIALS AND METHODS

### Cell line and vectors

The HCC cell line HuH7 was routinely maintained at our laboratory [35]. The 3 stable clones, HuH7-S2 (control), HuH7-core-high, and HuH7-core-low, were gifts kindly provided by Professor Jia-Horng Kao of National Taiwan University College of Medicine, Taipei, Taiwan. They were maintained in Dulbecco modified Eagle medium (DMEM) with 10% fetal bovine serum, 100 units/mL penicillin, 100 μg/mL streptomycin, 2 mM L-glutamine, and 25 ng/mL amphotericin B. For evaluating angiogenesis, human umbilical vein endothelial cells (HUVECs) were purchased from the Food Industry Research and Development Institute, Taiwan. They were maintained in an endothelial cell medium (ScienCell, Carlsbad, CA, USA). All cells were incubated at 37°C in a humidified incubator with 5% CO_2_.

### Tube formation assay

A transwell system was used for examining the interaction between HCC cell lines and HUVECs. The lower compartment of each 24-well transwell plate was coated with Matrigel. Each well was seeded with 4×10^4^ HUVECs in Medium 200PRF (Thermo Fisher, Waltham, MA, USA). The upper compartment of each transwell plate was seeded with 1×10^4^ HuH7-S2, HuH7-core-high, or HuH7-core-low cells in serum-free DMEM. The upper compartment was then placed onto the lower compartment and the transwell plates were incubated at 37°C in a 5% CO_2_ environment for 6 hours. Calcein AM fluorescent dye was added and incubation was continued for another 30 minutes. Next, the cells in the lower compartment were examined under a microscope and quantitatively analyzed using MetaMorph (Molecular Devices, Sunnyvale, CA, USA). For validating the effects of VEGF, 1 mg/mL bevacizumab (Roche, Switzerland) or control immunoglobulin G1 (IgG1) was added to the culture media of both the HCC cells and HUVECs. For validating the effects of AP-1 inhibition, 50 μM of an AP-1 inhibitor, T-5224 (Apexbio, Houston, TX, USA), was added to the culture media of both the HCC cells and HUVECs.

### Matrigel plug assay

Conditioned media were collected after the culturing of the HuH7-S2, HuH7-core-high, and HuH7-core-low cells in serum-free DMEM at a density of 5×10^6^ cells per 75T flask for 18 hours. The media were concentrated to 250 μL by using Amicon Ultra (Millipore, Billerica, MA, USA). Next, 600 μL of Matrigel was mixed with 90 μL of 1000 U/mL heparin and 45ng of recombinant mouse VEGF (positive control) (R&D, Minneapolis, MN, USA) or 100 μL of concentrated culture medium. The total volumes were equalized to 900 μL by adding DMEM. Matrigel plugs containing DMEM and heparin were only negative controls. The products were injected into the subcutaneous tissues of the abdomen of 5-week-old nude mice. After 7 days, the mice were sacrificed. The Matrigel plugs were homogenized and centrifuged. Next, 50 μL of the supernatant was mixed with 950 μL of Drabkin reagent, and the absorbance of the resultant solution at 540 nm was examined for hemoglobin content after 30 minutes. For validating the effects of VEGF, bevacizumab or control IgG1 was mixed with Matrigel to a final concentration of 1 mg/mL. Human VEGF (R&D) was used as a positive control instead because bevacizumab could not inhibit mouse VEGF. All animal experiments complied with the National Institutes of Health guide for the care and use of laboratory animals.

### Western blot analysis

All Western blot analyses were performed according to the standard Western blot protocols and antibody manufacturers’ instructions. Antibodies for the HCV core protein and VEGF were purchased from Abcam (Cambridge, UK), and antibodies for extracellular signal-regulated kinase (ERK), mitogen-activated protein kinase kinase (MEK), phosphorylated MEK, and cyclin D1 were purchased from Santa Cruz (Dallas, TX, USA). The antibody against β-actin was purchased from Sigma–Aldrich (St. Louis, MO, USA), and that against HIF-1α from EMD Millipore (Darmstadt, Germany). The remaining antibodies were purchased from Cell Signaling (Beverly, MA, USA).

### Quantitative reverse transcription PCR analysis

We performed quantitative reverse transcription PCR analysis according to the standard protocol by using SYBR Green (Roche, Mannheim, Germany). Glyceraldehyde-3-phosphate dehydrogenase (GAPDH) was used as the internal control. The sequences of primers are listed in the Supplementary Methods.

### Luciferase reporter assay

The protocols of the reporter assay kit (Qiagen, Valencia, CA, USA) and luciferase detection kit (Promega, Madison, WI, USA) were followed for detecting the transcriptional activity of AP-1 and STAT3, respectively. In Brief, cells were transfected with reporter plasmids (Qiagen) in 96-well plates and incubated for 48 hours. Dual–Glo® Luciferase Reagent (Promega) was added to the wells, and the luciferase activity of the experimental reporter was detected 10 minutes later. Subsequently, Dual-Glo® Stop & Glo® Reagent (Promega) was added to the wells, and the luciferase activity of the control reporter was detected 10 minutes later. The ratio of the luminescence of the experimental reporter to that of the control reporter was calculated. The effect of an AP-1 inhibitor, T-5224, was assessed by adding various concentrations of T-5224 to the cells 24 hours after transfection with the reporter plasmids and recording luciferase activity 48 hours later.

### Chromatin immunoprecipitation

After chromatin and protein were cross-linked, cells were lysed and harvested. We used sonication to shear DNA to fragments with approximately 500 base pairs. The protocols of the EZChIP kit (EMD Millipore) were closely followed. Mouse IgG (negative control) or antibodies against p-c-jun (Ser73) (Cell Signaling) were added for incubation overnight at 4°C. Protein G agarose was utilized to capture the antibody-protein-DNA complexes. After washing and reverse the crosslink, DNA was purified using a DNA purification kit (Qiagen). We then used quantitative PCR similar to the aforementioned methods to measure the DNA content. The sequences of the used primers are listed in the Supplementary Methods.

### Immunofluorescence staining

After fixation and permeabilization, HCC cells were stained with antibodies against HCV core protein (Abcam), p-c-jun (Cell Signaling) (Ser63) or p-c-fos (Ser32) (Cell Signaling) overnight at 4°C. An anti-rabbit polyclonal antibody (Thermo Fisher) was used as the secondary antibody. Nuclei were counterstained using 4’,6-diamidino-2-phenylindole (DAPI).

### RNA knockdown

Cells were seeded in 6-well plates at a density of 1.5×10^5^ cells. After 1:200 dilution of DharmaFECT 4 transfection reagent (GE Dharmacon, Lafayette, CO, USA) with culture medium, scrambled siRNA or siRNA against c-jun was added to the final concentration of 100nM. The cells were incubated with the mixture at 37°C.

### Immunohistochemistry studies of patient samples

Patients with an advanced HCC diagnosis from 2001 to 2011 with available tumor tissues obtained upon the diagnosis of advanced HCC at National Taiwan University Hospital (NTUH) were enrolled in this study. Patients with either HBV or HCV infection were included, whereas those with both HBV and HCV infection were excluded. The medical records of each patient were reviewed for confirming the tumor staging and history of hepatitis infection. This study was approved by the Research Ethical Committee of NTUH.

Tissue sections (thickness, 5 μm) were cut from formalin-fixed paraffin-embedded tissue blocks of tumors. Tissue sections were deparaffinized and autoclaved for 10 minutes in citrate buffer (pH 6.0) for antigen retrieval. The sections were incubated overnight at 4°C with primary antibodies against VEGF (1:2000, Santa Cruz, Santa Cruz, CA, USA) or p-c-jun (Ser63) (1:100, Cell Signaling). Signals were produced using appropriate secondary antibodies, followed by a horseradish peroxidase-labeled complex (Biocare Medical Starr trek HRP Universal Kit, Biocare, Taoyuan, Taiwan, R.O.C.) and a diaminobenzidine substrate. All slides were counterstained with hematoxylin and blindly reviewed by a single pathologist (M-S. Hsieh). Appropriate positive controls were stained simultaneously to ensure staining quality. We used two methods to assess VEGF staining. First, we analyzed it according to the staining intensity as strong or weak. Then the results of VEGF staining were assessed using the H score system. The H scores were calculated as the intensity (0, 1, 2, or 3) was multiplied by percentages of positively staining cells. High p-c-jun nuclear localization was defined as more than 50% of tumor cells with nuclear p-c-jun expression.

### Statistical analyses

Statistical analyses were performed using SAS statistical software (version 9.3; The SAS Institute, Cary, NC, USA). A 2-sided *p* value of ≤ 0.05 was considered statistically significant. We used the chi-square test (or Fisher exact test when suitable) for comparing the nominal IHC staining results (i.e. strong vs weak) between HBV- and HCV-related HCC. We used the independent t test to compare the mean H scores between HBV- HCC and HCV-related HCC.

## SUPPLEMENTARY MATERIALS FIGURES AND TABLE



## Abbreviations

HCC: hepatocellular carcinoma
HBV: hepatitis B virus
HCV: hepatitis C virus
HIF: hypoxia-inducible factor
JNK: Jun amino-terminal kinases
MAPK: mitogen-activated protein kinase
ANG: angiopoietin
VEGF: vascular endothelial growth factor
AP-1: activator protein-1
STAT3: signal transducer and activator of transcription 3
p-c-jun: phospho-c-jun
p-c-fos: phospho-c-fos
DMEM: Dulbecco modified Eagle medium
HUVEC: human umbilical vein endothelial cell
IgG1: immunoglobulin G1
ERK: extracellular signal-regulated kinase
MEK: mitogen-activated protein kinase kinase
GADPH: Glyceraldehyde-3-phosphate dehydrogenase
NTUH: National Taiwan University Hospital
