# Supplementary material for: Hepatitis C virus core protein potentiates proangiogenic activity of hepatocellular carcinoma cells
Source: Oncotarget. 2017 Sep 30;8(49):86681–92. doi: 10.18632/oncotarget.21407 (PMC5689717; doi:10.18632/oncotarget.21407)
Supplement: Supplementary file 1 [file oncotarget-08-86681-s001.pdf]

Hepatitis C virus core protein potentiates proangiogenic activity of hepatocellular carcinoma cells

SUPPLEMENTARY MATERIALS

SUPPLEMENTARY METHODS

Quantitative reverse transcription PCR analysis

We performed quantitative reverse transcription PCR analysis according to the standard protocol by using SYBR Green (Roche, Mannheim, Germany). Glyceraldehyde-3-phosphate dehydrogenase (GAPDH) was used as the internal control. The sequences of primers were 5′-AAGCGGCTGTACTGCAAAAAC (forward) and 5′-TTGATGTGAGGGTCGCTCTTC (reverse) for fibroblast growth factor-2, 5′-GGGCAGAATCATCACGAAGTG (forward) and 5′-CACCAGGGTCTCGATTGGAT (reverse) for VEGF, 5′-AGTTCCACCACCAAACATGCA (forward) and 5′-CACTATATGAAAATCCTGGCTCACA (backward) for VEGF-C, 5′-TGAGGGCTTTCGCCTTAGC (reverse) and 5′-CGGTAGTGAACCCGTTGATGT (reverse) for transforming growth factor (TGF)-β1, 5′-AAGAAGCAATATCAGGTCCAGCAT (forward) and 5′-GAGGAAGAGCGGCAGTTGTC (reverse) for ANG-2, 5′-AGCCAAAACGCCCCAAAC (forward) and 5′-TGTCATGCGTGTGCTTGAATT (reverse) for platelet derived growth factor (PDGF)-BB, and 5′-CCAAATCACCACCTGCTACACA (forward) and 5′-TCGTTCCAACAGCCAGTCTGT (reverse) for tissue inhibitor of metalloproteinase-4.

Chromatin immunoprecipitation (ChIP)

After chromatin and protein were cross-linked, cells were lysed and harvested. We used sonication to shear DNA to fragments with approximately 500 base pairs. The protocols of the EZChIP kit (EMD Millipore) were closely followed. Mouse IgG (negative control) or antibodies against p-c-jun (Cell Signaling) were added for incubation overnight at 4°C. Protein G agarose was utilized to capture the antibody-protein-DNA complexes. After washing and reverse the crosslink, DNA was purified using a DNA purification kit (Qiagen).

We then used quantitative PCR similar to the aforementioned methods to measure the DNA content. Primers for the 3 potential AP-1 binding sites were 5′-AAGATGTGGAGAGTTGGAGG (forward) with 5’-CCTGCGTGATGATTCAAACC (reverse), 5’-GCCCATTCCCTCTTTAGCC (forward) with 5’-AGCCGTTCCCTCTTTGCT (reverse), and 5’-GGAAACCAGCAGAAAGAGGA (forward) with 5’-GGGAGGAGGTGGTAGCTG (reverse). Primers targeting the promoter region not predictive as AP-1 binding sites were used as the control; their sequences were 5’- GAACTGCCTTCAGAGCCA (forward) and 5’-CACTTATCCTCACCTCCCTC (reverse). The quantity detected in samples without ChIP (input) was used as the reference.

Supplementary Table 1: Immunohistochemistry results

		N	VEGF H scores	*p* value
**Viral etiology**	HBV	94	248.8	< 0.001
	HCV	37	289.5	
**Phospho-c-jun**	Low	50	243.8	0.038
	High	72	261.2	

Abbreviations: HBV = hepatitis B virus; HCV = hepatitis C virus; VEGF = vascular endothelial growth factor.

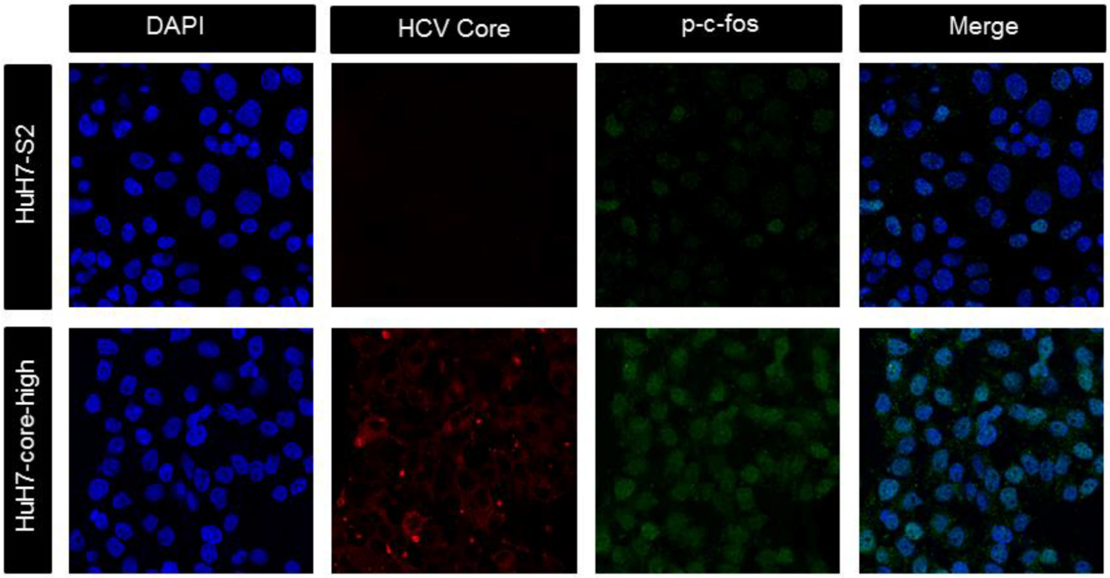

Supplementary Figure 1: Images (200×) of immunofluorescence staining results of HuH7-S2 and HuH7-core-high cells with DAPI for nuclear staining (blue) and antibodies against HCV core protein (red) and p-c-fos (green).

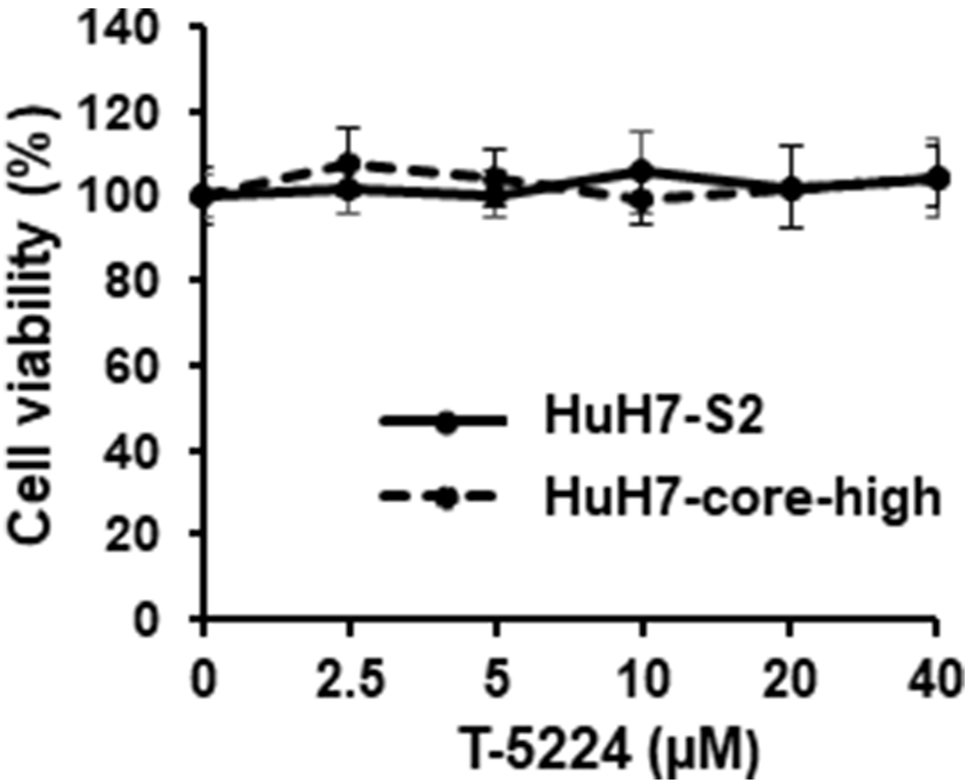

Supplementary Figure 2: A 3-(4,5-dimethylthiazol-2-yl)-2,5-diphenyltetrazolium bromide (MTT) assay examining the viability of HCC cell lines after treatment with the AP-1 inhibitor T-5224 at the indicated concentration for 72 hours.

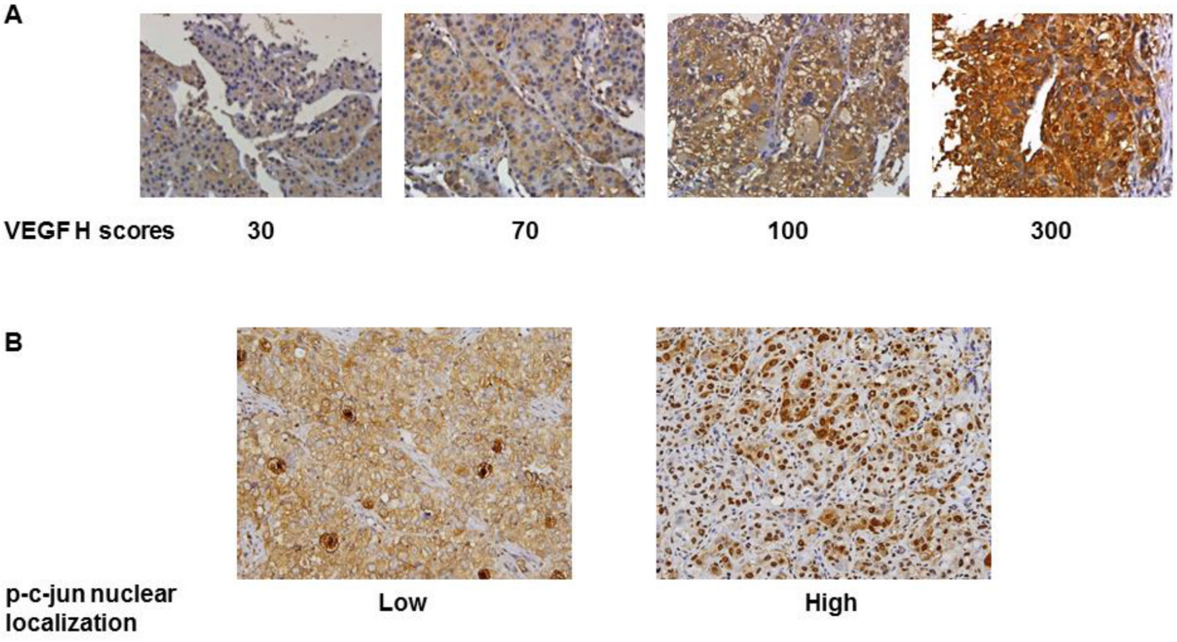

Supplementary Figure 3: Representative photos of immunohistochemical staining results of **(A)** VEGF with various H scores and **(B)** p-c-jun with or without nuclear localization.
